# Challenges of developing a cardiovascular risk calculator for patients with rheumatoid arthritis

**DOI:** 10.1371/journal.pone.0174656

**Published:** 2017-03-23

**Authors:** Cynthia S. Crowson, Silvia Rollefstad, George D. Kitas, Piet L. C. M. van Riel, Sherine E. Gabriel, Anne Grete Semb, On behalf of A Trans-Atlantic Cardiovascular Risk Consortium for Rheumatoid Arthritis (ATACC-RA)

**Affiliations:** 1 Division of Biomedical Statistics and Informatics, Department of Health Sciences Research and Division of Rheumatology, Department of Medicine, Mayo Clinic, Rochester, Minnesota, United States of America; 2 Preventive Cardio-Rheuma Clinic, Department of Rheumatology, Diakonhjemmet Hospital, Oslo, Norway; 3 Dudley Group NHS Foundation Trust, West Midlands, United Kingdom; 4 Department of Rheumatic Diseases, Radboud University Nijmegen Medical Centre, Nijmegen, Netherlands; University of Birmingham, UNITED KINGDOM

## Abstract

**Objective:**

Cardiovascular disease (CVD) risk calculators designed for use in the general population do not accurately predict the risk of CVD among patients with rheumatoid arthritis (RA), who are at increased risk of CVD. The process of developing risk prediction models involves numerous issues. Our goal was to develop a CVD risk calculator for patients with RA.

**Methods:**

Thirteen cohorts of patients with RA originating from 10 different countries (UK, Norway, Netherlands, USA, Sweden, Greece, South Africa, Spain, Canada and Mexico) were combined. CVD risk factors and RA characteristics at baseline, in addition to information on CVD outcomes were collected. Cox models were used to develop a CVD risk calculator, considering traditional CVD risk factors and RA characteristics. Model performance was assessed using measures of discrimination and calibration with 10-fold cross-validation.

**Results:**

A total of 5638 RA patients without prior CVD were included (mean age: 55 [SD: 14] years, 76% female). During a mean follow-up of 5.8 years (30139 person years), 389 patients developed a CVD event. Event rates varied between cohorts, necessitating inclusion of high and low risk strata in the models. The multivariable analyses revealed 2 risk prediction models including either a disease activity score including a 28 joint count and erythrocyte sedimentation rate (DAS28ESR) or a health assessment questionnaire (HAQ) along with age, sex, presence of hypertension, current smoking and ratio of total cholesterol to high-density lipoprotein cholesterol. Unfortunately, performance of these models was similar to general population CVD risk calculators.

**Conclusion:**

Efforts to develop a specific CVD risk calculator for patients with RA yielded 2 potential models including RA disease characteristics, but neither demonstrated improved performance compared to risk calculators designed for use in the general population. Challenges encountered and lessons learned are discussed in detail.

## Introduction

Patients with rheumatoid arthritis (RA) have a demonstrated increased risk of cardiovascular disease (CVD) of 1.5–2 fold compared to their peers without RA [1]. The elevated risk has been shown to be attributable to both traditional and RA-specific factors. Decision-making regarding indication for cardio-protective medication is supported by use of various CVD risk prediction algorithms. However, risk calculators designed for use in the general population do not accurately estimate the risk of CVD in patients with RA [2, 3].

By using risk prediction models, clinical care decisions are made every day based on the probability of disease or the probability of future events [4, 5]. The methodology for deriving and assessing risk calculators has received much attention in the statistical literature [6–10]. In fact, guidelines for reporting multivariable prediction models have recently been published [11].

Several attempts have been made to improve CVD risk prediction in patients with RA. The European League Against Rheumatism (EULAR) advocate to apply a 1.5 multiplier to the calculated risk by the nationally recommended CVD risk algorithm, QRISK2 have included a factor of 1.4 for patients with RA, and the Expanded cardiovascular Risk Score for RA (ERS-RA), which is the first published RA specific CVD risk calculator. However, these approaches had significant limitations [1, 12, 13]. A Trans-Atlantic Cardiovascular Consortium for Rheumatoid Arthritis (ATACC-RA) was formed in 2012 with the goal of building a risk calculator that would accurately predict risk of future CVD among patients with RA. This paper will describe general challenges involved in development of a risk calculator, and more specifically, bottlenecks in derivation of a risk calculator to optimize prediction of future CVD in RA patients.

## Methods

### Study populations

Thirteen cohorts of patients with RA originating from 10 different countries (UK, Norway, Netherlands, USA, Sweden, Greece, South Africa, Spain, Canada and Mexico) were combined for the purpose of developing an RA-specific CVD risk algorithm. Details regarding these cohorts are located in Table 1, on the consortium website (http://www.atacc-ra.com) and in previous publications [2, 3, 14–24]. The study was approved by ethical boards/committees at each center. These included the Mayo Clinic and Olmsted Medical Center Institutional Review Boards, CMO Arnhem Nijmegen, Dudley Local Research Ethics Committee, Office of the Oslo University Hospital's Privacy and Data Protection Officer, Ethics Committee at the University Hospital of Umeå, Harbor-UCLA Institutional Review Board, Laiko Hospital Institutional Review Board (Athens, Greece), Human Research Ethics Committee (Medical) from the University of the Witwatersrand in Johannesburg, South Africa, University of Manitoba Health Research Ethics Board and the Manitoba Health Information Privacy Committee, Ethics Committee of Cantabria for Hospital Universitario de Valdecilla in Santander (Spain), Comites de Ética e Investigación del Instituto Nacional de Ciencias Médicas y Nutrición Salvador Zubirán, and Arthritis Center Twente Institutional Review Board. The Mayo Clinic Institutional Review Board approved the cohort aggregation. Data were anonymized and aggregated for analyses.

**Table 1:** Characteristics of included cohorts.

Center	Name/ institution	Origin cohort	Name of Cohort	Cohort type (population based vs. referral)	Type of cohort (incident/Established	Age (years)	Disease duration at inclusion	Inclusion criteria for RA	Inclusion years	End Follow up
1	Mayo Clinic, Olmsted County, Minnesota	United States		Population-based	Incident and prevalent	54.7 (21, 91)	3.5 (0, 48)	1987 ACR criteria for RA	2000–2009	2014
2	Radboud University Medical Centre, Nijmegen	Netherlands		Population-based	Incident	54.8 (17, 87)	0.1 (0, 1)	1987 ACR criteria for RA and DMARD naïve	1985–2011	2011
3	Dudley	United Kingdom		Referral	Established	60.1 (26, 89)	12.4 (0, 48)	1987 ACR criteria for RA	2002–2008	2011
4	Diakonhjemmet Hospital, Oslo	Norway	The European Research on Incapacitating Diseases and Social Support: EURIDISS		Established in 1991	59.5 (32, 79)	12.2 (10, 15)		2002	2013
			The Oslo RA registry: ORAR		Established in 1994	58.2 (37, 79)	15.1 (13, 20)		2007	2013
5	Northern Sweden regional RA cohort, Umeå	Sweden		Population-based	Incident	54.2 (18, 87)	0.6 (0, 1.7)		1995–2003	2008
6	Harbor-UCLA Medical Center, Torrance, California	United States		Referral	Established	53.2 (22, 76)	10.7 (0.2, 37)		2010–2011	2013
7	Hospital Universitario Marqués de Valdecilla, Santander	Spain		Referral	Established	59.2 (19, 88)	7.8 (0, 40)		2010–2013	2014
8	Laikon Hospital, Athens	Greece		Referral	Established	57.7 (24, 80)	9.4 (0.2, 46)	1987 ACR criteria for RA	2009–2012	2013
9	Milpark Hospital Johannesburg	South Africa		Referral	Established	53.7 (24, 78)	10.8 (0.2, 56)	1987 ACR criteria for RA	2001–2012	2013
10	University of Manitoba, Winnipeg	Canada	Canadian Early Arthritis Cohort		New onset	48.6 (21, 79)	1.1 (0,8)	At least 1 inflamed joint	2000–2013	2013
11	Instituto Nacional de Ciencias Médicas y Nutrición Salvador Zubirán	Mexico		Consecutive patients seen at Early Arthritis Clinic	New Onset	37.6 (16, 79)	0.4 (0, 1)	Symptoms onset ≤12 months with no prior rheumatic diagnosis except RA (99% ACR criteria met)	2004–2013	2014
12	Brigham & Women’s hospital, Boston, Massachusetts	United States	Brigham and Women’s Rheumatoid Arthritis Sequential Study (BRASS)	Referral	Established	55.6 (18, 91)	12.5 (0, 58)	Rheumatologist-verified RA diagnosis	2003–2012	2014
13	Spectrum Twente, Enschede	Netherlands	Arthritis Cohort Twente Cardiovascular Disease (ACT-CVD)	Consecutive patients seen at a rheumatic outpatient clinic	New onset and Established	57.8 (20, 84)	6.8 (0, 53)	Rheumatologist-verified RA diagnosis	2009–2011	2012

### Variable definitions

#### Outcomes

The primary outcomes were fatal/non-fatal CVD events including acute coronary syndrome (ST-elevation and non-ST elevation myocardial infarction and unstable angina pectoris), chronic ischemic heart disease (stable angina pectoris), coronary revascularization (e.g., percutaneous coronary intervention and coronary artery bypass grafting), coronary death, other cardiovascular death, cerebrovascular events (ischemic cerebrovascular accident and transient ischemic attack) and peripheral vascular events (with and without revascularization procedures, peripheral artery disease). Not included were cases of confirmed cerebral hemorrhage, non-coronary cardiac death, non-ischemic cardiovascular disease, heart failure and aortic aneurysm.

#### Cardiovascular risk factors

Traditional CVD risk factors collected at baseline were: age, sex, smoking status (current, former, never), systolic and diastolic blood pressure, lipid levels (total cholesterol [TC], high density lipoprotein cholesterol [HDL-c], low density lipoprotein cholesterol [LDL-c], triglycerides, and TC:HDL-c ratio), body mass index (BMI), family history of CVD, diabetes mellitus and hypertension. The use of statins and antihypertensive medications at baseline were also registered.

#### RA disease characteristics

Clinical characteristics were collected at baseline including rheumatoid factor (RF) positivity, anti-citrullinated protein antibodies (ACPA) positivity, erythrocyte sedimentation rate (ESR), C-reactive protein (CRP), swollen and tender joint counts based on 28 joints, patient and physician global visual analog scales (VAS), disease activity score including 28 joints and ESR (DAS28ESR), RA disease duration and health assessment questionnaire (HAQ) disability index. RF and ACPA were considered positive based on the tests performed at each center. ACPA testing was lacking in some patients. CRP was not available from 1 cohort, DAS28ESR was not available from 1 cohort, swollen and tender joint counts were not available from 2 cohorts, patient global VAS was not available from 4 cohorts, physician global VAS was not available from 8 cohorts, and HAQ was not available from 3 cohorts. Data were also collected on antirheumatic medication use at baseline including synthetic and biologic disease modifying anti-rheumatic drugs (DMARDs) and corticosteroids.

### Statistical methods

Data from all cohorts were combined into one database. Patients with a history of CVD prior to baseline were excluded. Multiple imputation methods were used to impute missing values for the CVD risk factors using 10 repetitions. Two cohorts were missing a large amount (~75%) of lipid data, but the lipid data were mostly complete from the other cohorts, so only 20% of the lipid data was missing in the combined database. The missing lipid data were imputed, but analyses excluding these 2 cohorts were also performed to ensure the imputed data did not overly influence our results. Log-transformations were used when imputing lipid levels to avoid bias when computing lipid ratios from imputed data [25]. Descriptive statistics (means, percentages, etc.) were used to summarize the CVD risk factor and RA disease characteristic data and comparisons across cohorts were performed using chi-square and rank sum tests. Person-year methods were used to calculate the rate of CVD events for each cohort. Kaplan-Meier methods were used to plot the rates of development of CVD events during follow-up. Inverse probability weighting was used to adjust the CVD event rates for differences in CVD risk factors across the cohorts [26].

Cox proportional hazard regression analysis was used to develop the risk calculator model. Due to heterogeneity in CVD event rates between centers, the Cox models were stratified by center. To avoid issues with instability of estimates for small centers and to reduce the complexity of the model, centers were grouped into 2 strata (high and low CVD risk) based on CVD event rates. For patients with long follow-up, follow-up time was truncated at 12 years after baseline to facilitate accurate prediction of CVD risk at the 10 year time point, as truncating at ten years would necessitate predicting at the edge of the data and including data well beyond 10 years could influence the coefficients of the risk factors. Least Absolute Shrinkage and Selection Operator (LASSO)-penalized Cox regression was used for variable selection of CV risk factors [27]. Determination of the optimal model was based on cross-validation methods (using the minimal lambda minus 1 standard error rule to reduce the potential for overfitting). RA disease characteristics were then considered one-by-one for inclusion in the model following inclusion of the CVD adjustors because many of these measures were missing for 1 or more centers.

Smoothing splines were also used to examine potential non-linear effects of the continuous risk factors. No significant non-linear effects were noted for the CVD risk factors. Assumptions of proportional hazards were checked. Predicted probabilities for CVD were obtained from the final multivariable model.

Model performance was assessed with measures of discrimination and calibration using 10-fold cross validation. Discrimination was assessed using the concordance statistic (c-statistic) as adapted for the Cox model by Harrell [28]. The c-statistic is analogous to the area under the receiver operating characteristic (ROC) curve. Calibration was assessed by comparing observed versus expected events in deciles of predicted risk using goodness-of-fit tests similar to the Hosmer-Lemeshow test, as well as with standardized incidence ratios (SIR) (which compare the overall observed to predicted number of events) and with calibration plots [10]. Calculated predicted events were transformed to expected events [10]. An SIR>1 indicates that the observed events are higher than expected, meaning the risk calculator underestimates actual risk. Conversely, an SIR<1 indicates that the observed events are lower than expected, meaning that the risk calculator overestimates actual risk.

CVD risk was calculated for the purpose of comparison using risk algorithms designed for use in the general population, including Framingham risk score (FRS) for general CVD risk, American College of Cardiology/American Heart Association (ACC/AHA) Pooled Cohort Equation, QRISK2 and Systematic COronoary Risk Evaluation (SCORE) [5, 12, 29, 30]. Data on heart failure were not available in some cohorts, so the FRS predictions were reduced proportionately based on the fraction of non-heart failure events in the Framingham cohort to ensure a fair comparison between observed and expected events. For QRISK2, the Townsend deprivation score, atrial fibrillation and chronic kidney disease were not available in our data, so CVD risk was calculated using a modified QRISK2 algorithm excluding these variables. The outcome for SCORE includes only fatal events, which limited our analyses using SCORE due to the small number of fatal events in our cohort. Reclassification was examined, but the net reclassification index was not computed, as its use is not recommended when miscalibration exists, which is the case for centers in our low CVD risk strata [9, 31]. Analyses were performed using SAS version 9.4 (SAS Institute, Cary, NC, USA) and R 3.2.0 (R Foundation for Statistical Computing, Vienna, Austria).

## Results

### Baseline characteristics

A total 5638 RA patients without prior CVD were included (mean age: 55 [SD: 14] years, 76% female; Table 2). During a mean follow-up of 5.8 years (30139 person years), 389 patients developed a CVD event. Two cohorts consisted of Hispanics (US/UCLA and Mexico), the rest were almost exclusively Caucasians. RA disease duration varied by cohort: 4 with early RA (<1 year), 7 established RA (mean 9–13 years) and 2 with both. All demographics, RA disease characteristics and CVD risk factors differed significantly between cohorts (p<0.001 for all).

**Table 2:** Descriptive baseline characteristics of 5638 patients with rheumatoid arthritis without prior cardiovascular disease, overall along with lowest and highest mean/percentage by cohort.

Characteristic	Available N	Total (N = 5638)	Cohort variation	
			Lowest mean/percentage	Highest mean/percentage
Length of follow-up, years	5638	5.8 (4.4)	1.6	9.5
Calendar year of RA diagnosis	5628	1998.4 (9.9)	1990.4	2006.8
Age, years	5685	55.3 (14.0)	37.6	60.1
Sex, female	5638	4278 (76%)	66%	90%
White race	3945	3699 (94%)	63%	100%
***CVD risk factors***				
Systolic blood pressure, mmHg	5345	136.0 (21.9)	115.6	147.6
Diastolic blood pressure, mmHg	5343	79.6 (11.1)	72.0	85.7
Total cholesterol, mmol/L	4457	5.2 (1.1)	4.4	5.7
Low density lipoprotein cholesterol, mmol/L	4364	3.1 (1.0)	2.3	3.9
High density lipoprotein cholesterol, mmol/L	4403	1.5 (0.4)	1.1	1.7
Triglycerides, mmol/L	4240	1.4 (0.8)	1.1	1.7
Current smoker	5368	1148 (21%)	7%	33%
Ever smoker	5120	2688 (52%)	16%	74%
Body mass index, kg/m^2^	5160	27.0 (5.4)	24.9	29.0
Hypertension	5609	2344 (42%)	9%	67%
Diabetes mellitus	5637	395 (7%)	3%	17%
Family history of CVD	3677	876 (24%)	3%	50%
***RA disease characteristics***				
RA disease duration, years	5628	6.4 (9.2)	0.1	12.9
RF and/or ACPA positive	5485	3949 (72%)	50%	92%
Erythrocyte sedimentation rate, mm/hr	4737	24.8 (21.4)	15.4	32.5
C-reactive protein, mg/L	4528	15.4 (27.1)	2.0	28.3
Severe extra-articular manifestations of RA	4795	262 (6%)	0%	17%
DAS28ESR	4448	4.0 (1.7)	2.5	6.0
Swollen joint count 28	4267	6.3 (6.2)	1.7	13.5
Tender joint count 28	4267	6.2 (6.6)	1.6	13.8
Patient VAS	3851	39.0 (25.7)	31	56
HAQ	3192	0.7 (0.7)	0.2	1.5
***Medications***				
Antihypertensive medication	5608	1314 (23%)	1%	42%
Lipid-lowering medication	5604	510 (9%)	0%	41%
Synthetic DMARD use	5593	2610 (47%)	0%	100%
Biologic DMARD use	5592	891 (16%)	0%	60%
Corticosteroid use	5590	1527 (27%)	8%	73%
Corticosteroid dosage, mg/day	869	5.8 (4.4)	4.5	9.1

Values in table are mean (SD) or n (%). Abbreviations: RA = rheumatoid arthritis, CVD = cardiovascular disease, RF = rheumatoid factor, ACPA = anti-citrullinated protein antibody, DAS = disease activity score, ESR = erythrocyte sedimentation rate, HAQ = health assessment questionnaire, DMARD = disease modifying anti-rheumatic drug

### Primary outcome

**Fig 1:**
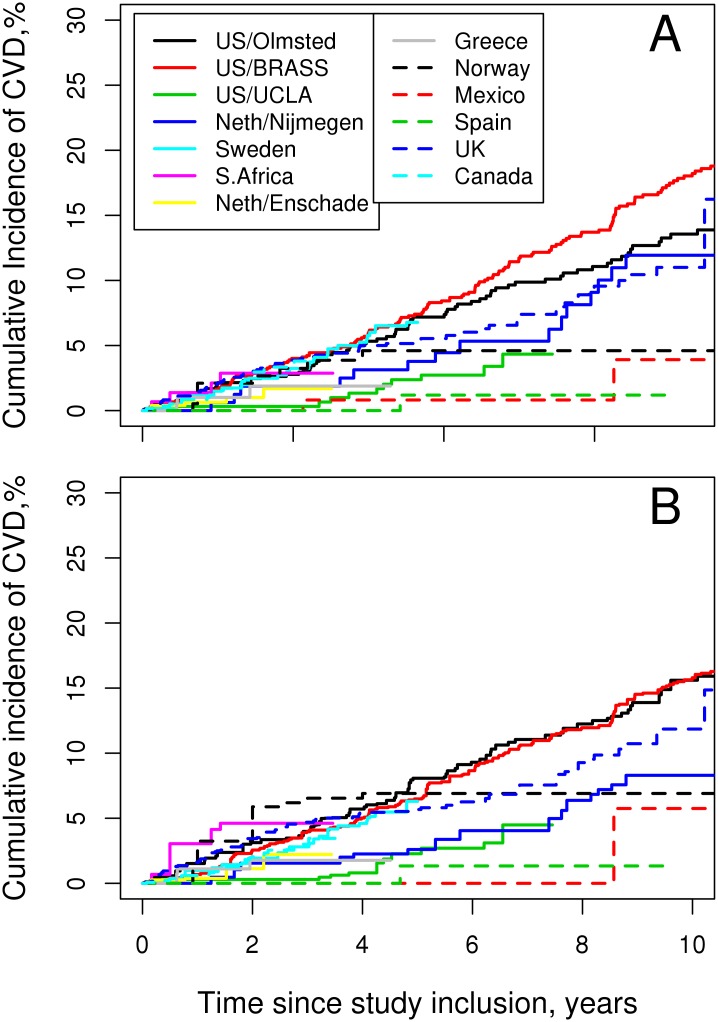
Cardiovascular event rates by cohort. (A) unadjusted rates. (B) rates adjusted for cardiovascular risk factors.

CVD event rates varied across countries (range 0.1–1.8%/year) with the lowest observed in Canada, Mexico and UK and the highest in US, Netherlands, and Sweden (Table 3 and Fig 1A). Adjusting for CVD risk factors to account for the differences between cohorts modified the CVD event rates, but did not reduce the heterogeneity in CVD event rates across cohorts (Fig 1B). Due to heterogeneity in CVD event rates between centers, risk calculator models were stratified by high and low CVD risk center groups with Netherlands (Nijmegen and Enschede), US (Olmsted and Brigham and Women’s Rheumatoid Arthritis Sequential Study) and Sweden in the high CVD risk strata and all the other centers in the low CVD risk strata.

**Table 3:** Cardiovascular Disease (CVD) event rates by cohort (ordered by CVD event rate).

cohort	n	CVD events	Mean length of follow-up (95% CI)	Person-years of observation	CVD event rate (%/ yr.)
Netherlands/Nijmegen	1049	147	7.8 (0.9, 12.0)	8226.5	1.8
Sweden	404	31	4.8 (1.8, 5.0)	1936.0	1.6
US/Olmsted	661	76	8.1 (0.7, 12.0)	5322.6	1.4
Netherlands/Enschede	585	22	3.0 (1.4, 3.5)	1741.8	1.3
US/BRASS	1090	65	4.9 (0.5, 10.1)	5380.4	1.2
Norway/EURIDISS-ORAreg	161	15	8.4 (3.4, 11.1)	1349.8	1.1
US/UCLA	147	4	2.7 (0.3, 3.4)	398.2	1.0
South Africa	191	8	5.2 (1.0, 10.6)	995.7	0.8
Greece	197	3	2.2 (1.0, 3.8)	427.6	0.7
Spain	509	5	1.6 (0.2, 2.7)	808.4	0.6
UK	313	10	5.9 (1.2, 7.3)	1844.6	0.5
Canada	186	2	5.0 (0.2, 11.8)	930.3	0.2
Mexico	145	1	5.4 (0.3, 9.6)	777.5	0.1
OVERALL	5638	389	5.3 (0.5, 12.0)	30139.3	1.3

Abbreviations: BRASS = Brigham and Women’s Rheumatoid Arthritis Sequential Study, UCLA = University of California Los Angeles

### Risk model development

The next step of the risk calculator development involved assessment of CVD risk factors. CVD risk factors considered for inclusion were: age, sex, current smoker, ever smoker, systolic and diastolic blood pressure measures (in mmHg and log-transformed), lipid measures (TC, HDL-c, LDL-c, triglycerides, and TC:HDL-c ratio), BMI, family history of CVD, diabetes mellitus, history of hypertension, use of anti-hypertensives and use of anti-lipemic medications. The optimal model based on cross-validation methods included 5 variables (age, sex, presence of hypertension, current smoker and TC:HDL-c ratio) consistently across all 10 imputation sets. Inclusion of the presence of hypertension instead of systolic blood pressure was undesirable because there is a loss of information in the dichotomous hypertension variable compared to the continuous blood pressure measure. However, inexplicably, systolic and diastolic blood pressure values were not significant predictors of CVD events in this RA cohort.

Adjusting for the CVD risk factors, each RA disease characteristics was assessed individually for potential inclusion in the risk calculator (Table 4). Several RA disease characteristics were significant predictors of CVD (i.e., DAS28ESR, swollen and tender joint counts, both patient and physician VAS and HAQ). Several other RA disease related characteristics only approached statistical significance. The multivariable modeling revealed 2 possible risk calculators including either DAS28ESR or HAQ along with age, sex, presence of hypertension, current smoker and TC:HDL-c ratio (Table 5). Additional RA disease characteristics did not add significantly to these models and non-linear effects were not identified.

**Table 4:** Assessment of rheumatoid arthritis disease characteristics in cardiovascular disease risk score model. Each potential risk factor is added to a model including age, sex, current smoking, hypertension and lipid ratio (total cholesterol: high density lipoprotein cholesterol).

Characteristic	Hazard ratio (95% CI)
RF positivity	1.18 (0.94, 1.47)
ACPA positivity	0.98 (0.78, 1.23)
RF/ACPA positivity	1.14 (0.90, 1.45)
ESR (linear), per 10 mm/hr	1.03 (0.99, 1.08)
ESR (log-transformed)	1.05 (0.94, 1.19)
CRP (linear), per 10 mg/L	1.03 (0.99, 1.06)
CRP (log-transformed)	1.05 (0.98, 1.13)
DAS28ESR	1.13 (1.05, 1.22)
Swollen joint count	1.03 (1.01, 1.05)
Tender joint count	1.03 (1.02, 1.05)
Patient global VAS, per 10 mm	1.12 (1.06, 1.17)
Physician global VAS, per 10 mm	1.15 (1.05, 1.26)
RA disease duration, per 10 years	0.92 (0.82, 1.04)
HAQ (linear)	1.37 (1.14, 1.64)

Abbreviations: CI = confidence interval; RF = rheumatoid factor; ACPA = anti-citrullinated protein antibodies; ESR = erythrocyte sedimentation rate; CRP = C-reactive protein; DAS = disease activity score; VAS = visual analog scale; HAQ = health assessment questionnaire disability index

**Table 5:** Coefficients and hazard ratios of risk calculator models.

Variable	Model A: Risk score with DAS			Model B: Risk score with HAQ		
	Coefficient	Hazard ratio	p-value	Coefficient	Hazard ratio	p-value
Age (per 10 years)	0.482	1.62	<0.001	0.473	1.60	<0.001
Sex (male)	0.479	1.61	<0.001	0.476	1.61	<0.001
Current smoking	0.416	1.52	0.002	0.419	1.52	0.005
Hypertension	0.531	1.70	<0.001	0.640	1.90	<0.001
TC:HDL lipid ratio	0.340	1.40	0.010	0.416	1.52	0.003
DAS28ESR	0.126	1.13	0.001			
HAQ				0.313	1.37	<0.001
Baseline survival at 10 years*						
High risk centers	0.921			0.916		
Low risk centers	0.963			0.968		

Abbreviations: TC = total cholesterol; HDL = high-density lipoprotein cholesterol; DAS = disease activity score; ESR = erythrocyte sedimentation rate; HAQ = health assessment questionnaire disability index

*for age = 55.3, male = 0, current smoker = 0, hypertension = 0, TC: HDL = 2.17, DAS28ESR = 4.0, HAQ = 0.73

### Model performance

**Fig 2:**
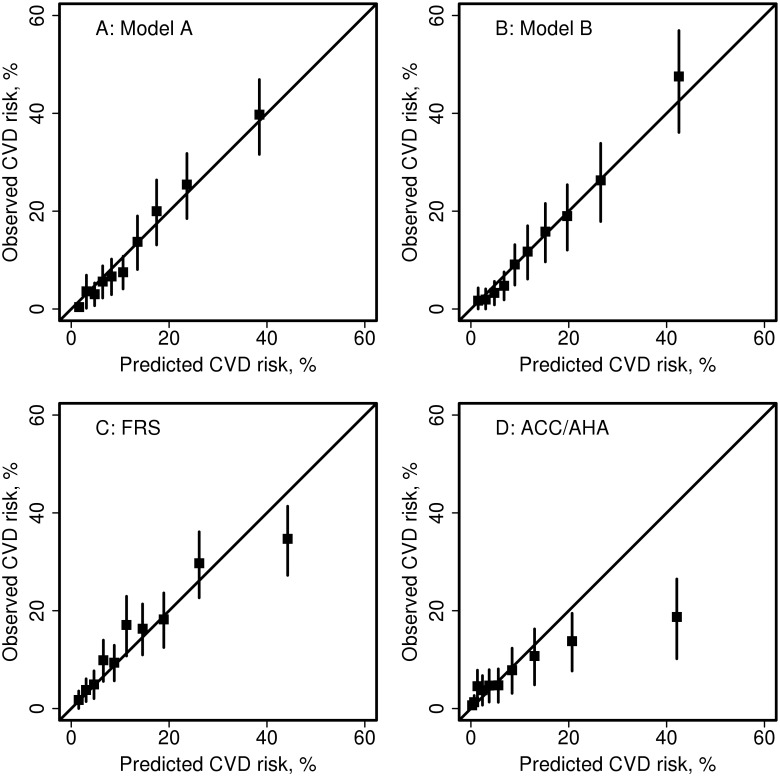
Calibration plots: Observed versus predicted 10-year risk for Cardiovascular Disease (CVD) according to deciles of predicted risk. (A) Model A, (B) Model B, (C) the Framingham risk score (FRS) and (D) the American College of Cardiology/American Heart Association Pooled Cohort Equation (ACC/AHA). The vertical lines are 95% confidence intervals for the observed CVD risk estimates.

The developed risk calculators demonstrated discrimination that was no better than the established risk calculators (c-statistic: 0.71 for FRS, 0.72 for ACC/AHA, 0.70 for SCORE and 0.72 for QRISK2). Ten-fold cross-validation was used to provide an internal validation, and it revealed c-statistics of 0.70 for Model A and 0.71 for Model B (Table 6). Overall calibration of the developed risk calculators assessed using ten-fold cross-validation yielded SIR = 0.83 for both models, indicating that the risk calculator estimates were significantly higher than observed events. Moderate calibration, assessed according to deciles of predicted risk, was better in the developed risk calculators than in the established risk calculators as none of the deciles demonstrated significant differences between observed and predicted risks in the 2 new risk calculators (Fig 2). In contrast, both the FRS and the ACC/AHA underestimated CVD risk in the highest decile of predicted risk. The ACC/AHA also overestimated CVD risk in several other deciles of predicted risk. SCORE was not depicted because the smaller number of fatal events in our cohort made it difficult to assess deciles of predicted risk. However, SCORE significantly overestimated the observed fatal CVD events in our cohort (SIR: 0.43) QRISK2 also substantially overestimated the observed risk of CVD in nearly all of the deciles (SIR: 0.60).

**Table 6:** Performance of developed risk calculators compared with general CVD risk calculators.

Performance measure	Model A**	Model B**	FRS	ACC/AHA	SCORE	QRISK2
Discrimination						
C statistic (95% CI)	0.70 (0.66–0.74)	0.71 (0.67–0.75)	0.71 (0.67–0.74)	0.72 (0.69–0.75)	0.70 (0.67–0.74)	0.72 (0.69–0.76)
Calibration						
Decile-based test, p-value*	0.23	0.31	<0.001	<0.001	<0.001	<0.001
Standardized incidence ratio (95% CI)	0.83 (0.74–0.94)	0.83 (0.73–0.94)	1.04 (0.94–1.15)	1.02 (0.90, 1.16)	0.43 (0.30–0.60)	0.60 (0.54–0.67)

Abbreviations: CI = confidence interval, FRS = Framingham risk score, SCORE = Systematic COronary Risk Evaluation, ACC/AHA = American College of Cardiology/American Heart Association

*Adaptation of Hosmer-Lemeshow test for use in survival analyses. A significant value indicates deviations between observed and predicted outcomes. Models A and B tested with 8 instead of 9 degrees of freedom to account for evaluation in the dataset used to fit the model

**Model performance assessed using 10-fold cross validation to avoid over-optimism

### Reclassification of risk estimates

**Fig 3:**
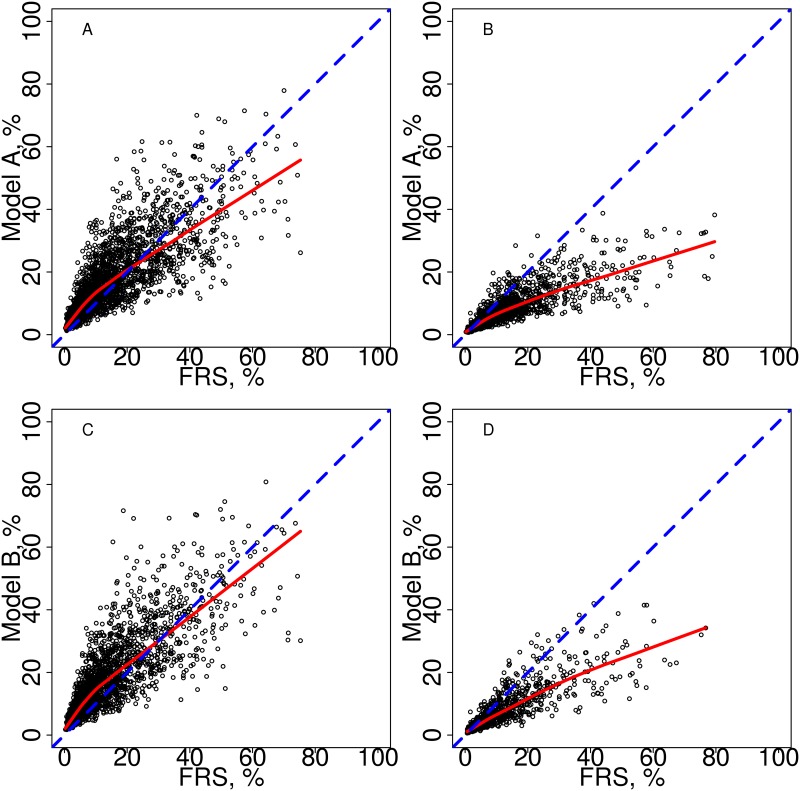
Comparison of the developed risk calculators (Model A and Model B) to the Framingham Risk Score (FRS). (A) Comparing Model A and FRS for patients in the high cardiovascular risk strata. (B) Comparing Model A and FRS for patients in the low cardiovascular risk strata. (C) Comparing Model B and FRS for patients in the high cardiovascular risk strata. (D) Comparing Model B and FRS for patients in the low cardiovascular risk strata. In each panel, the dashed line represents identity and the solid line is a smoother line of trend.

Scatter plot comparisons of the calculated risk of CVD by the developed risk calculators compared to the calculated risk by FRS demonstrated that many of the new risk calculator values were lower than the FRS values in the high risk centers and the majority of the new risk calculator values were lower than the FRS values in the low risk centers (Fig 3).

Reclassification was assessed separately for patients in the high and low CVD risk centers (Table 7). In the high CVD risk centers, 46% of patients remained in the same CVD risk category when classified by Model A as when classified by FRS, 36% of patients were classified into a higher risk category by Model A than by FRS and 8% of patients were classified into a lower risk category by Model A than by FRS. In the low CVD risk centers, 53% of patients remained in the same CVD risk category when classified by Model A as when classified by FRS, only 2% of patients were classified into a higher risk category by Model A than by FRS and 45% of patients were classified into a lower risk category by Model A than by FRS. In both the high and low risk centers, patients with ≥20% CVD risk calculated by FRS who were classified to 10-<20% risk by Model A did not demonstrate substantially lower observed CVD rates (21.6% for high risk centers and 19.9% for low risk centers), calling into question the accuracy of reclassification to lower risk by Model A. The observed event rates in the other re-categorized patients agreed with the new classification by Model A in most cases, but the confidence intervals were wide due to small numbers of patients with events in many categories. Reclassification was not examined for SCORE and QRISK2 due to their extremely poor calibration in our cohort.

**Table 7:** Reclassification for centers with high and low CVD risk for Model A compared with Framingham Risk Score (FRS).

**High CVD risk Centers**			**Model A**			
			<5%	5- <10%	10- <20%	≥ 20%
**FRS**	<5%	N w/o events	329	320	52	1
		N w/ events	5	9	4	0
		Observed 10 yr risk (95% CI)	2.5 (0.0, 5.3)	3.6 (1.2, 6.0)	9.8 (0.0, 19.1)	0.0
	5- <10%	N w/o events	15	241	289	35
		N w/ events	0	9	22	7
		Observed 10 yr risk (95% CI)	0.0	6.4 (1.3, 11.3)	12.1 (6.8, 17.1)	30.8 (7.8, 48.0)
	10- <20%	N w/o events	1	64	371	203
		N w/ events	0	4	32	30
		Observed 10 yr risk (95% CI)	0.0	9.1 (0.0, 18.5)	16.4 (10.2, 22.1)	26.1 (15.4, 35.6)
	≥ 20%	N w/o events	0	3	107	424
		N w/ events	0	0	12	108
		Observed 10 yr risk (95% CI)	0.0	0.0	21.6 (7.3, 33.6)	36.5 (29.6, 42.7)
**Low CVD risk Centers**			**Model A**			
			<5%	5- <10%	10- <20%	≥ 20%
**FRS**	<5%	N w/o events	414	19	0	0
		N w/ events	1	1	0	0
		Observed 10 yr risk (95% CI)	0.3 (0.0, 0.9)	5.0 (0.0, 14.1)	0.0	0.0
	5- <10%	N w/o events	163	176	12	0
		N w/ events	1	7	2	0
		Observed 10 yr risk (95% CI)	2.6 (0.0, 7.4)	6.3 (1.4, 11.0)	0.0	0.0
	10- <20%	N w/o events	29	247	139	2
		N w/ events	2	9	3	0
		Observed 10 yr risk (95% CI)	0.0	6.5 (1.9, 10.9)	13.8 (0.0, 33.7)	0.0
	≥ 20%	N w/o events	0	52	173	67
		N w/ events	0	0	9	7
		Observed 10 yr risk (95% CI)	0.0	0.0	19.9 (3.5, 31.9)	25.4 (4.2, 41.9)

## Discussion

Efforts to develop a specific CVD risk calculator for patients with RA yielded 2 potential models including RA disease characteristics, but neither demonstrated improved performance compared to CVD risk calculators designed for use in the general population. While it is statistically feasible to develop an RA specific CVD risk calculator by pooling resources from many centers, its ability to optimize prediction of future CVD in this patient population is questionable due to several challenges.

### Population of interest

It is important that the data used to develop a risk calculator is representative of the patients who will be assessed by the risk calculator. For this purpose, population-based data may be preferred to ensure that the entire spectrum of CVD risk is represented in the data. Referral bias can have an unpredictable impact on CVD risk rates. For example, patients with RA have a high burden of disease that includes increased risks for multiple comorbidities, such as interstitial lung disease, osteoporosis, infections, hematologic cancers, as well as psychosocial impairments (e.g., fatigue, depression, and cognitive decline) [32]. Patients with RA referred to a cardiologist for CVD risk assessment may encompass a broad spectrum including both the high CVD risk patients with several traditional CVD risk factors and the patients whose RA and other comorbidities are controlled well enough that they will not be overwhelmed by information about their increased risk of CVD. Thus referral bias may lead to either over or under estimation of the true CVD risk in patients with RA. We aimed to develop a trans-Atlantic CVD risk calculator, which may have failed, among other factors, due to geographical differences. The countries included in our cohort have different healthcare systems, with differing access to health care, which may lead to differences in CVD risk [33]. In fact, countries with lower socioeconomic welfare tend to have delays in diagnosis of RA and stricter eligibility criteria for biologic therapies [34]. This inequity in access to treatment may influence disease activity levels, the ability to achieve remission and other long-term outcomes of patients with RA [35]. However, additional biases may also be at play, as the CVD risk estimated in our low CVD risk strata is substantially lower than the CVD event rates in the general population of the countries of origin. Perhaps optimal treatment of CVD risk factors and/or the RA disease activity with use of biologics and tight disease control resulting in remission has reduced the CVD event rate in these cohorts. Alternatively, there could be ascertainment bias leading to under-reporting of CVD events. Either way, it is unlikely that the low CVD event rates predicted by our risk calculator will provide accurate estimates of CVD risk for all patients with RA in the countries that had very low CVD risk rates in our cohort, since our calculator re-classified 45% of patient in the low risk centers to lower risk categories than the FRS calculator.

### Purpose of risk prediction

Many CVD risk calculators were designed to help determine which patients should receive lipid-lowering therapies, such as statins [5]. Due to this goal, historic cohorts with data collection prior to the availability of statins are often used to develop the risk calculators. These cohorts have the advantages of long follow-up and do not have any biases related to statin use. However, the CVD event rates in these cohorts may not reflect current CVD event rates, as there have been many advances in addition to statins that have improved CVD outcomes in recent years [36]. Another option would be to use more contemporary data and exclude (i.e., censor) subjects at the time when statins were initiated, but this could equate to systematic exclusion of higher risk patients, which would result in unrealistic CVD event rates. Our pooled cohort includes data from after the introduction of statins, but we did not make any adjustments for use of statins during follow-up, as these data were not available in all cohorts.

### Study design

Another important consideration is the study design. Longitudinal cohort studies and registries are most often used for developing risk calculators for long-term outcomes. These observational studies are a more practical and cost-effective way to obtain long-term follow-up than randomized clinical trials. However, observational studies suffer from confounding that can be difficult or impossible to address. For example, event rates from the past may not apply to contemporary patients due to changes in treatments, medical care or patient characteristics (e.g., obesity). Another source of confounding in observational studies is confounding by indication. In patients with RA, those with more severe disease are more likely to receive biologics. If biologics have an impact on CVD risk, this could bias the results of CVD risk assessment in patients with RA. The alternative to observational studies is randomized clinical trials. While randomized trials have the advantage of reducing confounding, they also have limitations. Importantly, patients with comorbidities are often excluded in randomized trials, so the trial population is unlikely to be representative of the population of interest for CVD risk evaluation in RA.

### Risk factor selection

Risk calculators are predicated on the notion that multiple risk factors may interact to increase the risk of developing a disease. Patients with modest increases in several risk factors may be at an equivalent or higher risk of disease than patients with one highly elevated risk factor. Therefore, clinical management decisions should account for the constellation of risk factors in order to optimize preventive strategies. Traditional CVD risk factors include age, sex, hypertension, hyperlipidemia, smoking and diabetes mellitus. Considerations for adding other risk factors for calculation of total CVD risk include assessments of the incremental value with regard to improvement in risk predictions, as well as the cost of recording of the risk factor in terms of both money and patient and clinician time/effort. DAS28 and HAQ were both considered and included in the risk prediction models developed in our study. HAQ is obtained from a short patient questionnaire, but DAS28 requires a professional joint assessment as well as an inflammatory marker, which make it a bit more time-consuming and expensive to measure than many of the traditional CVD risk factors.

In addition, causality is important, as risk factors included in a risk calculator are assumed to result in lower risk when they are improved. However, causality is difficult to prove except in randomized clinical trials, which are currently lacking regarding preventive interventions and CVD outcome in patients with RA. Yet promising results from post hoc analyses from 2 large randomized placebo controlled trials with hard CVD endpoints (the IDEAL and TNT trials) showed that patients without and with inflammatory joint diseases, including patients with RA, had comparable lipid lowering effects and reduction in CVD events after intensive treatment with statins [37].

There is a lack of optimal biomarkers to track RA disease activity. RF and ACPA are measures of disease severity, not disease activity. ESR and CRP are markers of inflammation, which correlate with disease activity. However, CRP has been shown to be a mediator, not a cause, of CVD in Mendelian randomization studies [38]. Swollen and tender joint counts and patient and physician global VAS are measures of disease activity, which have been incorporated into composite disease activity measures, such as the DAS28 and Clinical Disease Activity Index, although these measures are somewhat subjective [39, 40]. In addition, disease activity fluctuates over time in patients with RA, so it may not be reasonable to expect a single disease activity measure to accurately predict 10-year CVD risk. Indeed, cumulative measures of disease activity have shown stronger associations with CVD risk than single measurements among patients with RA [41, 42]. However, cumulative measures are impractical to implement in a risk calculator due to the difficulties of ascertaining them in clinical practice. Therapies for RA are also tempting to consider as risk factors for CVD in patients with RA, but their use is confounded by indication in observational studies. High dose glucocorticoids are thought to increase CVD risk, but low dose glucocorticoids may reduce CVD risk by controlling inflammation [43]. Methotrexate and biologic response modifiers may also modulate the risk of CVD, but causal associations have not been proven [44, 45]. These issues may partially explain why our risk prediction models did not perform better than CVD risk calculators for the general population, which do not incorporate RA-specific factors.

### Sample size considerations

Risk calculator development requires a large dataset with a high number of events. Obtaining a large sample size for a study of patients with RA can be difficult. Despite the fact that RA is one of the more common rheumatologic diseases, its prevalence is only 1%. Single centers typically have too few patients to develop risk calculators for patients with RA. However, pooling data from multiple centers may have challenges. Retrospectively collected data is often used to avoid waiting for follow-up to accrue in a prospective study. Despite predefined variables it may be difficult to ensure that study-specific factors (e.g., variable definitions, surveillance methods, identification of subjects, participation rates, etc.) are similar across centers. Registries often include larger numbers of patients than single center studies and are thought to be less heterogeneous due to standardized data collection procedures, but selection bias for enrolled subjects is an important issue that is often ignored. Heterogeneity can make it difficult or even impossible to develop a risk calculator depending on whether the sources of the heterogeneity can be adequately explained and counteracted or not. Our study found significant heterogeneity between centers, which was addressed by including low and high risk strata in our risk prediction models.

### Performance assessment

The performance of a risk calculator requires assessment of both discrimination and calibration. Discrimination is correctly ranking patients from low to high risk. It is commonly assessed using the area under the receiver operating characteristic curve, which is also known as the c-statistic. The c-statistic has a small practical range from a minimum of 0.5 (i.e., no predictive ability) to a hypothetical maximum of about 0.88. This narrow range has led to criticism of the c-statistic as a useful measure of the incremental value of new risk factors [46]. The c-statistics of our models were not better than the c-statistics for the general population risk calculators.

Calibration is accurately predicting the absolute risk level. It is often assessed using the Hosmer-Lemeshow goodness-of-fit test for binary outcomes or analogous variations for time-to-event outcomes [10]. Van Calster et al define 4 levels of calibration including: 1) mean calibration (i.e., calibration-in-the-large; or simply that the average predicted risk is similar to the average observed risk), 2) weak calibration (i.e., a calibration intercept of zero and a calibration slope of one; or simply that there is no systematic over or under estimation of predicted risks), 3) moderate calibration (i.e., among patients with the same predicted risk, the observed risk equals the predicted risk, so among patients with a 10% predicted risk, 10% will have an observed event) and 4) strong calibration (i.e., predicted risks and observed event rates correspond for every covariate pattern) [8]. Strong correlation is desirable but unrealistic and may lead to overly complex models, so moderate calibration has been recommended. Graphical assessments of calibration are often more useful than statistical tests [6]. While our risk prediction models showed better calibration than for the general population risk calculators, this could reflect the fact that re-calibration is often needed when applying a risk calculator to a different population and calibration is expected to be better in the cohort used to develop the model than in other cohorts.

All risk calculators should be validated prior to adoption for clinical use. Validation can be done internally or externally. Internal validation can be performed by dividing the original cohort into derivation and test sets or by using cross-validation techniques. We used 10-fold cross-validation to assess the performance of our risk calculator models, in order to avoid the over-optimism associated with assessing a model in the same data that was used to develop it. Unfortunately, this internal validation did not show improved performance of our models compared to general population risk calculators.

The lack of improvement in CVD risk assessment demonstrated by our models is consistent with the recent findings of Alemao et al that adding CRP to the FRS or QRISK2 did not improve CVD risk assessment for patients with RA [47]. Similarly, the Reynolds risk score, which includes CRP, did not demonstrate improved performance for CVD risk assessment among patients with RA [2] Since the increased risk of CVD among patients with RA likely stems from the systemic inflammation that characterizes RA, adding an inflammatory marker to a CVD risk calculator seems reasonable, but it has not proven to be effective. As mentioned previously, this lack of improvement may stem from the inherent fluctuations in disease activity over time in patients with RA, so it may not be reasonable to expect a single disease activity measure to accurately predict 10-year CVD risk. Alternatively, RA therapies may be altering CVD risk. Since most patients with RA will change treatments several times during a 10 year period, it could be difficult to accurately estimate 10-year CVD risk without knowing which treatments the patient will receive in the future. Therefore, accurate assessment of CVD risk in patients with RA is complex.

## Conclusion

Our risk calculators did not demonstrate improved performance among patients with RA compared to general population CVD risk calculators. There are many issues involved in derivation of a risk calculator, which make development of a CVD risk calculator for use in patients with RA particularly challenging. These challenges should be considered by others who may attempt to make a CVD risk calculator for patients with RA and by those who seek to develop risk calculators for other purposes in other patient populations. In addition to the challenges in development of a risk calculator, there may be certain obstacles in implementation of such a calculator in clinical practice. A risk calculator that includes both cardiovascular and rheumatologic factors might require coordinated care from both a rheumatologist and a cardiologist or general practitioners to obtain all the measures needed to assess it, particularly if joint counts were required. Even patient-reported measures like the HAQ require time for calculation [48].

Finally, the true worth of a risk calculator depends on its ability to improve health outcomes. However, few studies have been done in the general population to prove that the use of a CVD risk calculator results in improved outcomes compared to single factor based approaches, despite the fact that the utility of risk calculators is assumed by several treatment guidelines for CVD risk prevention [5, 49, 50]. Among patients with RA, traditional CVD risk factors cannot explain all of the increased risk for CVD. RA disease specific factors have also been shown to play a role in the increased CVD risk, but it is not known whether optimal control of inflammation and RA disease activity would reduce the risk of CVD to that of the general population [51, 52]. Further research is needed to determine the best approaches to mitigating the increased risk of CVD among patients with RA. Meanwhile, clinicians should follow guidelines for CVD risk assessment in the general population and make sure that traditional CVD risk factors are optimally treated in their patients with RA.
