# Correction: Challenges of developing a cardiovascular risk calculator for patients with rheumatoid arthritis

**DOI:** 10.1371/journal.pone.0175605

**Published:** 2017-04-07

**Authors:** Cynthia S. Crowson, Silvia Rollefstad, George D. Kitas, Piet L. C. M. van Riel, Sherine E. Gabriel, Anne Grete Semb, on behalf of A Trans-Atlantic Cardiovascular Risk Consortium for Rheumatoid Arthritis (ATACC-RA)

The in-figure legend of Fig 1 appears incorrectly in the published article. Please see the correct Fig 1 and its caption here.

**Fig 1:**
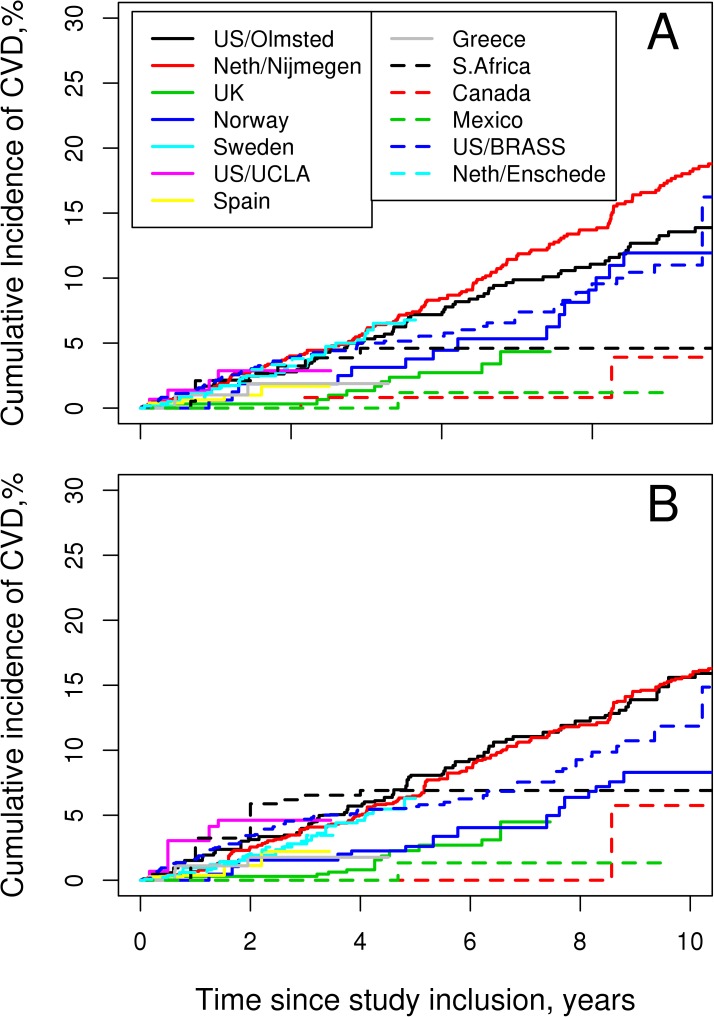
Cardiovascular event rates by cohort. (A) unadjusted rates. (B) rates adjusted for cardiovascular risk factors.
